# Transcriptomic analysis reveals prolonged neurodegeneration in the hippocampus of adult C57BL/6N mouse deafened by noise

**DOI:** 10.3389/fnins.2024.1340854

**Published:** 2024-02-12

**Authors:** Sang-Youp Lee, Ho Sun Lee, Min-Hyun Park

**Affiliations:** ^1^Department of Otorhinolaryngology, Seoul National University College of Medicine, Seoul, Republic of Korea; ^2^Department of Otorhinolaryngology, Boramae Medical Center, Seoul Metropolitan Government-Seoul National University, Seoul, Republic of Korea

**Keywords:** noise-induced hearing loss, transcriptomics, neurodegeneration, cochlea, auditory cortex, hippocampus

## Abstract

**Introduction:**

Several studies have reported a significant correlation between noise-induced hearing loss and cognitive decline. However, comprehensive analyses of this relationship are rare. This study aimed to assess the influence of hearing impairment on cognitive functions by analyzing organ samples in the afferent auditory pathway of deafened mice using mRNA sequencing.

**Methods:**

We prepared 10 female 12-week-old C57BL/6N mice as the experimental and control groups in equal numbers. Mice in the experimental group were deafened with 120 dB sound pressure level (SPL) wideband noise for 2 h. Cochlea, auditory cortex, and hippocampus were obtained from all mice. After constructing cDNA libraries for the extracted RNA from the samples, we performed next-generation sequencing. Subsequently, we analyzed the results using gene ontologies (GOs) and Kyoto Encyclopedia of Genes and Genomes (KEGG) pathway databases for differentially expressed genes (DEGs) of each organ.

**Results:**

Our results revealed 102, 89, and 176 DEGs for cochlea, auditory cortex, and hippocampus, respectively. We identified 294, 203, and 211 GOs; 10, 7, and 17 KEGG pathways in the cochlea, auditory cortex, and hippocampus, respectively. In the long term (12 weeks) from noise-induced hearing loss, GOs and KEGG pathways related to apoptosis or inflammation persisted more actively in the order of hippocampus, auditory cortex, and cochlea.

**Discussion:**

This implies that the neurodegenerative effects of noise exposure persist more longer time in the central regions.

## Introduction

Dementia is one of the biggest global healthcare problems, affecting 55 million people worldwide (Gauthier et al., 2022). Since there is no effective disease-modifying treatment option for dementia progression (Tisher and Salardini, 2019), the prevention of early-stage cognitive decline seems more important. Notably, it has been reported that hearing loss is associated with dementia (Lin et al., 2011; Gurgel et al., 2014). As relevant studies progressed, hearing loss has been accepted as a major modifiable risk factor for dementia (Livingston et al., 2017). It is thought that hearing loss can lead to cognitive decline, which may then serve as a “second hit,” exacerbating dementia in the presence of organic brain pathology (Lin and Albert, 2014).

Several independent studies have suggested underlying mechanisms by which hearing impairment leads to cognitive decline. Some studies suggest that the increased cognitive demands resulting from hearing loss can evoke cognitive decline (Lin and Albert, 2014; Cardin, 2016). Other researchers suggest that the oxidative stress triggered by hearing loss may adversely affect the hippocampus by increasing neuroinflammation, accelerating apoptosis, and reducing neurogenesis (Gonzalez-Perez et al., 2011; Paciello et al., 2023). Abnormalities in the neurotransmitter system, such as the N-methyl-d-aspartic acid receptor 2B, have been reported to be related to cognitive decline (Cui et al., 2013). Disorders of Schaffer collateral-1 long-term potentiation through depressed levels of brain-derived neurotrophic factors due to high-intensity sound are known to cause cognitive decline (de Deus et al., 2021). Additionally, it has been proposed that stress hormones and corticosteroids produced in response to hearing loss-induced stress can impact neuronal function in the hippocampus, ultimately leading to cognitive decline (Jin et al., 2017; Kurioka et al., 2021).

These studies provide an overview of how hearing impairment affects cognitive function. However, it is difficult to draw a comprehensive explanation because each study reveals only discrete aspects of the phenomenon from the researcher's viewpoint. In this case, omics studies can provide a broader understanding. An omics study quantitatively analyzes the whole set of specific biomolecules, such as the genome, transcriptome, proteome, or metabolome, in a given time (Vailati-Riboni et al., 2017; Subedi et al., 2022). This enables us to obtain a balanced insight into complex genetic mechanisms. Gene expression patterns can be primarily understood by mRNA sequencing.

Recent studies using omics technologies have uncovered the hidden mechanisms of hearing loss progresses at the molecular level. A previous study showed that noise exposure immediately evokes global cochlear protein ubiquitylation and upregulates ribosomal proteins in the cochlea (Jongkamonwiwat et al., 2020). Another study found that acoustic trauma modulates the expression of inflammation- and immunity-related genes (Maeda et al., 2021; Miao et al., 2021). However, few omics studies have investigated the relationship between hearing loss and cognitive decline. A transcriptome-wide association study has shown that age-related hearing loss impairs the glutamatergic synapse pathway in the hippocampus of BXD-recombinant inbred mice (Deng et al., 2021). Except this, we could not find out any omics study on the relationship between hearing loss and cognitive decline.

From this perspective, the current study was designed to evaluate the impact of auditory impairment on cognitive functioning. This was achieved by analyzing tissue samples from key components of the afferent auditory pathway—specifically the cochlea, the auditory cortex, and the hippocampus—through mRNA sequencing techniques. The auditory cortex was selected for its critical role in the processing and interpretation of auditory signals. The hippocampus was included due to its integral function in spatial and episodic memory; noteworthily, hippocampal impairment has been identified as a hallmark feature in a range of cognitive disorders, including dementia (Nadhimi and Llano, 2021; Billig et al., 2022). Furthermore, we used gene ontologies (GOs) and the Kyoto Encyclopedia of Genes and Genomes (KEGG) pathway databases to examine the types of events that occur after noise-induced hearing loss.

## Materials and methods

### Animal management

Ten female C57BL/6N mice were used. To prevent sex act as a confounding factor, we used only female mice. Mice were placed in cage with freely access to distilled water and chow under pathogen free condition (12 h light/dark cycle, 23°C, 50% humidity). They were divided into experimental (*n* = 5) and control (*n* = 5) groups. Mice in the experimental group were deafened with a single exposure of 120 dB sound pressure level (SPL) wideband noise for 2 h in an audiometric booth at the age of 12 weeks. To confirm the audiometric state, we checked the hearing thresholds of both ears just before noise exposure, 2 weeks post-exposure, and at the age of 24 weeks just before sacrifice.

All experimental procedures were approved by the Institutional Animal Care and Use Committee (IACUC) of Boramae Hospital (IACUC number 2022-0133).

### Tissue preparation

All the mice were sacrificed at 24 weeks of age. They were fully anesthetized by isoflurane inhalation in an airtight cage. Cardiac perfusion was performed using phosphate-buffered saline (PBS) to remove red blood cells from the tissues. After decapitation, we harvested the bilateral cochleae, auditory cortical areas, and hippocampus of each mouse by referencing an anatomy atlas (Franklin and Paxinos, 2013). All right and left specimens were combined into one sample for each mouse organ.

### mRNA sequencing

The samples were lysed using the RNeasy Mini Kit (Qiagen, Germany) following the manufacturer's instructions for total RNA extraction. Total RNA quantity and quality were determined by UV/Vis spectrophotometer (NanoDrop 2000, ThermoFisher Scientific, USA). All RNA samples showed suitable A260/A280 and 28s/18s ratios. A cDNA library was subsequently constructed, and next-generation sequencing was performed. The entire NGS procedure was performed using an Illumina NovaSeq 6000 system (Macrogen, South Korea).

### Statistical analysis on expressed genes

To reduce bias in the results, we conducted quality control analysis of the raw sequencing reads. This involved removing low-quality data, adaptor sequences, contaminant DNA, PCR duplicates, and other artifacts. After confirming all samples satisfied quality control criteria, we selected three sets out of five sets in analyzing samples of cochlea, auditory cortex, and hippocampus for experimental and control groups.

The preprocessed reads were then mapped to the reference genome using HISAT2 version 2.1.0 (https://ccb.jhu.edu/software/hisat2/index.shtml/) to generate aligned reads. Subsequently, transcript assembly was performed using StringTie version 2.1.3b (https://ccb.jhu.edu/software/stringtie/). Expression profiles were extracted through transcript quantification for each sample, and fragments per kilobase of transcript per million mapped reads (FPKM), reads per kilobase of transcript per million mapped reads (RPKM), and transcripts per kilobase million (TPM) values were calculated. Differentially expressed genes (DEGs) were selected using this process, and functional annotation and gene-set enrichment analyses were performed using the GOs and KEGG databases. We set |fold change| of ≥2.0 and *p*-value of < 0.05 as statistically significant. For the selection of differentially expressed genes (DEGs), we utilized the raw *p*-values obtained from the direct gene comparisons between control and experimental groups. In contrast, for the identification of GOs and KEGG pathways, we employed adjusted *p*-values derived from hypergeometric testing and multiple testing corrections to control the false discovery rate. The sample size was determined to ensure sufficient quantity, taking into account cell type, tissue specificity, and library preparation methods. Since all samples met the quality control standards, we required only three samples per group.

We used GOnet (https://tools.dice-database.org/GOnet/) and REVIGO (http://revigo.irb.hr/) to visualize the relationships among genes and their ontologies. In the GOnet, we performed “GO term annotation” analysis using “generic GO slim” subset. In the REVIGO, we set resulting list size as “medium,” and chose “SimRel” semantic similarity measure.

## Results

### Audiometric tests

Prior to noise exposure, all the mice exhibited normal hearing thresholds in both ears during the auditory brainstem response (ABR) tests using click, 8 and 16 kHz tone-burst sound. Two weeks after noise exposure, it was found that none of the mice in the experimental group had an ABR response to sound stimulations of 90 dB SPL in either ear. In contrast, all the mice in the control group maintained normal thresholds in both ears. The hearing status of all mice was assessed prior to sacrifice, and it was confirmed that there had been no changes since the last hearing test.

### Data quality control

We excluded genes that were not detected in at least one of the 18 samples. Of the 45,777 genes detected, 27,122 were not detected in at least one sample and were excluded. Thus, 18,655 genes were analyzed in this study.

To reduce systemic bias in sample comparisons, we performed relative log-expression normalization prior to statistical analysis. The necessary size factor was estimated using the read count data. Additionally, we performed multidimensional scaling and hierarchical clustering analyses to check for the presence of outlier samples and similarity in expression patterns among biological replicates and confirmed that there were no discrepancies.

### Differentially expressed genes

**Figure 1:**
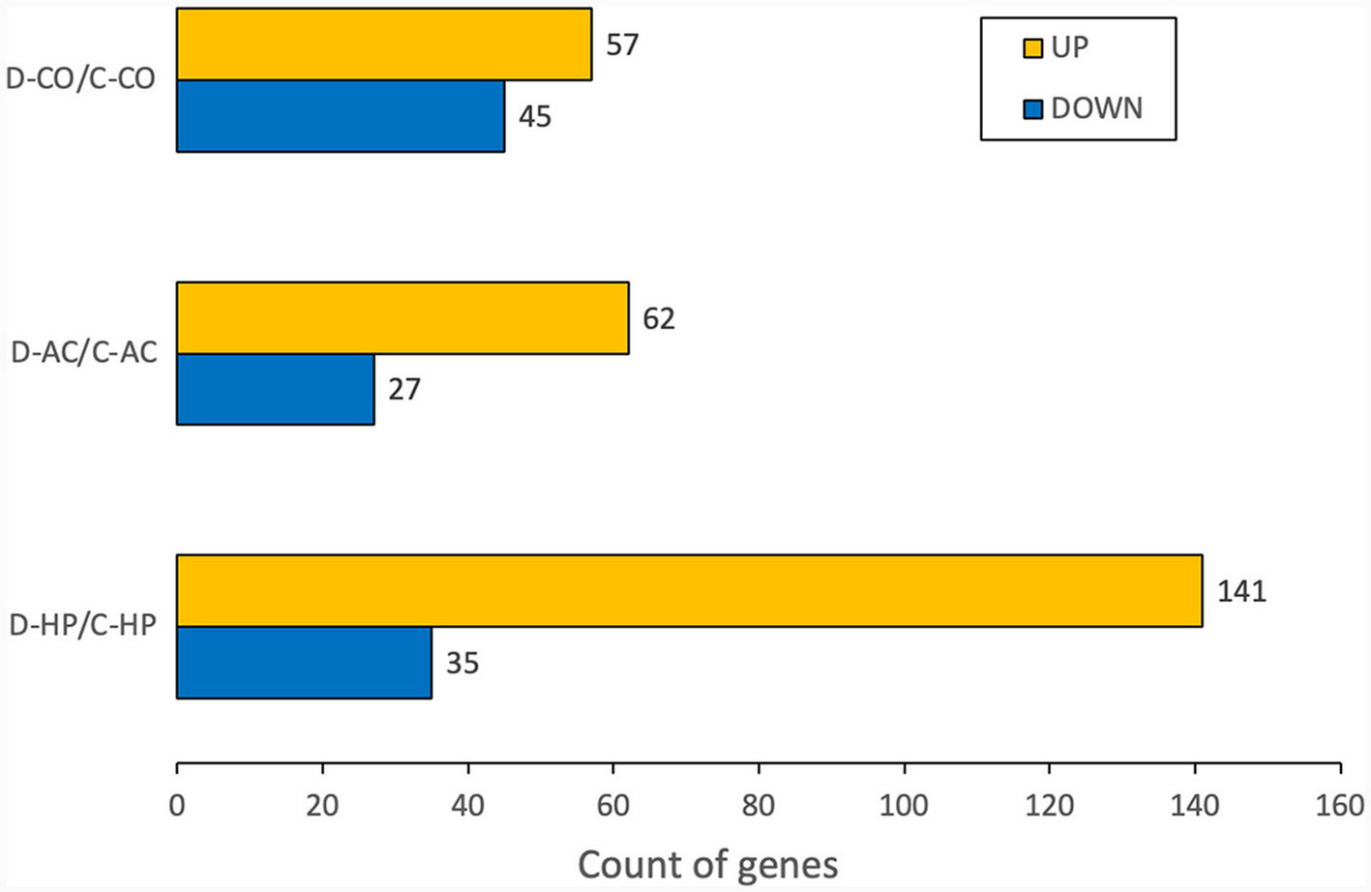
Number of DEGs for each organ. The numbers are calculated for the genes whose fold changes are >2 and raw *p*-values are within 0.05.

To compare the experimental and control groups, we used the up-regulated and down-regulated genes that were differentially expressed in each organ. In the cochlea, there were 57 up-regulated and 45 down-regulated DEGs, while in the auditory cortex, there were 62 up-regulated and 27 down-regulated DEGs. In the hippocampus, 141 DEGS were up-regulated, and 35 were down-regulated (Figure 1).

**Figure 2:**
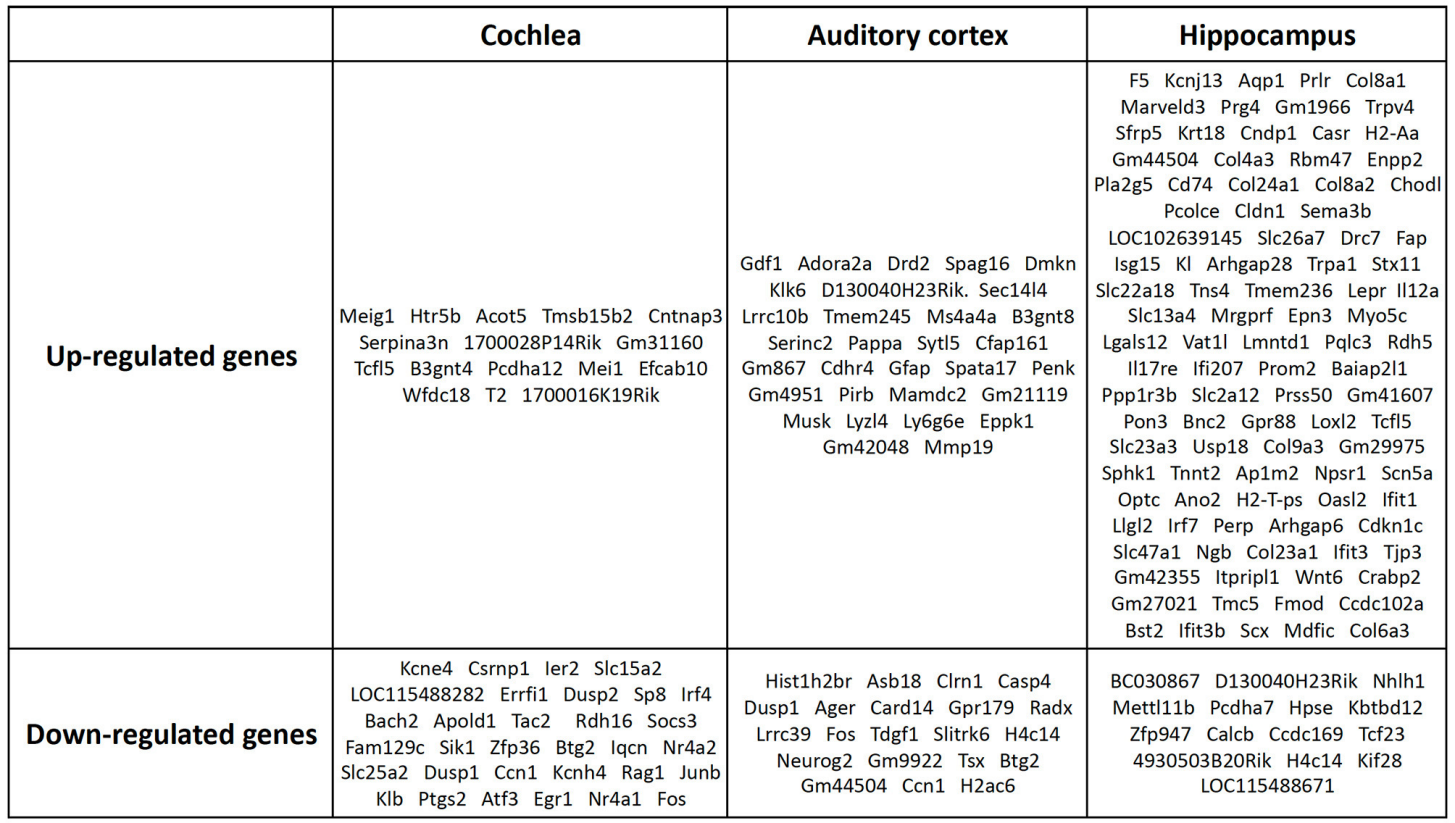
Protein-coding DEGs of the cochlea, audigory cortex, and hippocampus. In the cochlea, there were 16 up-regulated and 32 down-regulated protein-coding DEGs, while in the auditory cortex, there were 31 up-regulated and 21 down-regulated protein-coding DEGs. In the hippocampus, 99 up-regulated and 15 down-regulated protein-coding DEGs were expressed.

However, these results included not only protein-coding genes but also pseudogenes, lncRNAs, snoRNAs, and miRNAs. We excluded the non-protein-coding genes to determine the functions of the RNAs. When only the protein-coding genes were considered, the number of DEGs reduced. In the cochlea, there were 16 up-regulated and 32 down-regulated protein-coding DEGs (Table 1), while in the auditory cortex, there were 31 up-regulated and 21 down-regulated protein-coding DEGs (Table 2). In the hippocampus, 99 up-regulated and 15 down-regulated protein-coding DEGs were expressed (Table 3). In total, there are 146 up-regulated protein-coding DEGs and 68 down-regulated protein-coding DEGs (Figure 2). This transcriptomic data can be accessed in SRA database of NCBI site (reference number: PRJNA1061000).

**Table 1:** Differentially expressed genes in the cochlea.

**Gene ID**	**Gene symbol**	**Description**	**Fold change (deaf/control)**	**Raw *p*-value (deaf/control)**
**a. Up-regulated genes**				
104362	Meig1	Meiosis expressed gene 1	2.72451	0.00115
15564	Htr5b	5-hydroxytryptamine (serotonin) receptor 5B	2.55739	0.00696
217698	Acot5	Acyl-CoA thioesterase 5	2.53157	0.00317
100034363	Tmsb15b2	Thymosin beta 15b2	2.48637	0.00089
238680	Cntnap3	Contactin associated protein-like 3	2.47875	0.00331
20716	Serpina3n	Serine (or cysteine) peptidase inhibitor, clade A, member 3N	2.45358	0.00000
67483	1700028P14Rik	RIKEN cDNA 1700028P14 gene	2.42483	0.01373
102633301	Gm31160	Predicted gene, 31160, transcript variant X5	2.38870	0.00004
277353	Tcfl5	Transcription factor-like 5 (basic helix-loop-helix)	2.32988	0.00345
231727	B3gnt4	UDP-GlcNAc:betaGal beta-1,3-N-acetylglucosaminyltransferase 4	2.30858	0.00169
192164	Pcdha12	Protocadherin alpha 12	2.27350	0.00193
74369	Mei1	Meiotic double-stranded break formation protein 1	2.20177	0.00046
75040	Efcab10	EF-hand calcium binding domain 10	2.17077	0.00534
14038	Wfdc18	WAP four-disulfide core domain 18	2.13102	0.01006
21331	T2	Brachyury 2	2.12875	0.00004
74230	1700016K19Rik	RIKEN cDNA 1700016K19 gene	2.09831	0.00397
**b. Down-regulated genes**				
57814	Kcne4	Potassium voltage-gated channel, Isk-related subfamily, gene 4	−2.02804	0.01304
215418	Csrnp1	Cysteine-serine-rich nuclear protein 1	−2.04434	0.00124
15936	Ier2	Immediate early response 2	−2.05776	0.00506
57738	Slc15a2	Solute carrier family 15 (H+/peptide transporter), member 2	−2.06791	0.00000
115488282	LOC115488282	Uncharacterized LOC115488282, transcript variant X1	−2.08478	0.00443
74155	Errfi1	ERBB receptor feedback inhibitor 1	−2.10587	0.00413
13537	Dusp2	Dual specificity phosphatase 2	−2.12057	0.00014
320145	Sp8	Trans-acting transcription factor 8	−2.12946	0.02599
16364	Irf4	Interferon regulatory factor 4	−2.13488	0.00023
12014	Bach2	BTB and CNC homology, basic leucine zipper transcription factor 2	−2.14126	0.00001
381823	Apold1	Apolipoprotein L domain containing 1	−2.16870	0.03508
21334	Tac2	Tachykinin 2	−2.17265	0.00348
19683	Rdh16	Retinol dehydrogenase 16	−2.19381	0.01938
12702	Socs3	Suppressor of cytokine signaling 3	−2.24148	0.00516
100037278	Fam129c	Family with sequence similarity 129, member C	−2.27732	0.00000
17691	Sik1	Salt inducible kinase 1	−2.29470	0.00112
22695	Zfp36	Zinc finger protein 36	−2.44989	0.00618
12227	Btg2	B cell translocation gene 2, anti-proliferative	−2.45579	0.00008
637079	Iqcn	IQ motif containing N	−2.46003	0.01062
18227	Nr4a2	Nuclear receptor subfamily 4, group A, member 2	−2.52660	0.00069
83885	Slc25a2	Solute carrier family 25 (mitochondrial carrier, ornithine transporter) member 2	−2.74525	0.03672
19252	Dusp1	Dual specificity phosphatase 1	−2.80397	0.00000
16007	Ccn1	Cellular communication network factor 1	−2.86252	0.01801
380728	Kcnh4	Potassium voltage-gated channel, subfamily H (eag-related), member 4	−2.86412	0.00003
19373	Rag1	Recombination activating 1	−2.97390	0.00000
16477	Junb	Jun B proto-oncogene	−3.01155	0.00040
83379	Klb	Klotho beta	−3.17551	0.01020
19225	Ptgs2	Prostaglandin-endoperoxide synthase 2	−3.25476	0.00418
11910	Atf3	Activating transcription factor 3	−3.60211	0.00123
13653	Egr1	Early growth response 1	−4.08707	0.00151
15370	Nr4a1	Nuclear receptor subfamily 4, group A, member 1	−5.27951	0.00002
14281	Fos	FBJ osteosarcoma oncogene	−8.35415	0.00000

**Table 2:** Differentially expressed genes in the auditory cortex.

**Gene ID**	**Gene symbol**	**Description**	**Fold change (deaf/control)**	**Raw *p*-value (deaf/control)**
**a. Up-regulated genes**				
14559	Gdf1	Growth differentiation factor 1	16.52491	0.00010
11540	Adora2a	Adenosine A2a receptor	3.56210	0.00062
13489	Drd2	Dopamine receptor D2	3.51378	0.00426
66722	Spag16	Sperm associated antigen 16	3.07310	0.01554
73712	Dmkn	Dermokine	2.73336	0.00029
19144	Klk6	Kallikrein related-peptidase 6	2.71373	0.00722
211135	D130040H23Rik	RIKEN cDNA D130040H23 gene	2.69533	0.00246
103655	Sec14l4	SEC14-like lipid binding 4	2.60984	0.00482
278795	Lrrc10b	Leucine rich repeat containing 10B	2.56226	0.00062
242474	Tmem245	Transmembrane protein 245	2.42146	0.00380
666907	Ms4a4a	Membrane-spanning 4-domains, subfamily A, member 4A	2.40839	0.00888
232984	B3gnt8	UDP-GlcNAc:betaGal beta-1,3-N-acetylglucosaminyltransferase 8	2.33381	0.00027
230779	Serinc2	Serine incorporator 2	2.33204	0.00005
18491	Pappa	Pregnancy-associated plasma protein A	2.33128	0.01320
236643	Sytl5	Synaptotagmin-like 5	2.30804	0.00168
75556	Cfap161	Cilia and flagella associated protein 161	2.30577	0.00229
333670	Gm867	Predicted gene 867	2.25836	0.01556
69398	Cdhr4	Cadherin-related family member 4	2.24019	0.00583
14580	Gfap	Glial fibrillary acidic protein	2.17004	0.00029
74717	Spata17	Spermatogenesis associated 17	2.14429	0.04395
18619	Penk	Preproenkephalin	2.13625	0.01126
240327	Gm4951	Predicted gene 4951	2.13048	0.04154
18733	Pirb	Paired Ig-like receptor B	2.09880	0.00292
71738	Mamdc2	MAM domain containing 2	2.07577	0.00016
100861668	Gm21119	Predicted gene, 21119	2.05938	0.04798
18198	Musk	Muscle, skeletal, receptor tyrosine kinase	2.05913	0.00261
69032	Lyzl4	Lysozyme-like 4	2.05672	0.01272
70274	Ly6g6e	Lymphocyte antigen 6 complex, locus G6E	2.05624	0.00089
223650	Eppk1	Epiplakin 1	2.05213	0.01360
105246824	Gm42048	Predicted gene, 42048	2.01962	0.03546
58223	Mmp19	Matrix metallopeptidase 19	2.01409	0.00299
**b. Down-regulated genes**				
665622	Hist1h2br	Histone cluster 1 H2br	−2.00065	0.03103
208372	Asb18	Ankyrin repeat and SOCS box-containing 18	−2.00749	0.03440
229320	Clrn1	Clarin 1	−2.03603	0.00422
12363	Casp4	Caspase 4, apoptosis-related cysteine peptidase	−2.04280	0.01282
19252	Dusp1	Dual specificity phosphatase 1	−2.04784	0.00063
11596	Ager	Advanced glycosylation end product-specific receptor	−2.05349	0.00429
170720	Card14	Caspase recruitment domain family, member 14	−2.13673	0.02004
217143	Gpr179	G protein-coupled receptor 179	−2.16473	0.00713
102871	Radx	RPA1 related single stranded DNA binding protein, X-linked	−2.18001	0.00881
109245	Lrrc39	Leucine rich repeat containing 39	−2.28429	0.00044
14281	Fos	FBJ osteosarcoma oncogene	−2.30223	0.04435
21667	Tdgf1	Teratocarcinoma-derived growth factor 1	−2.37906	0.04659
239250	Slitrk6	SLIT and NTRK-like family, member 6	−2.42304	0.00278
97122	H4c14	Histone cluster 2, H4	−2.43449	0.02622
11924	Neurog2	Neurogenin 2	−2.67322	0.00954
102633345	Gm9922	Predicted gene 9922	−2.73599	0.00258
22127	Tsx	Testis specific X-linked gene	−2.83551	0.01123
12227	Btg2	B cell translocation gene 2, anti-proliferative	−3.00434	0.00000
100169864	Gm44504	Predicted readthrough transcript (NMD candidate), 44504	−3.62548	0.04615
16007	Ccn1	Cellular communication network factor 1	−3.63716	0.00380
319164	H2ac6	Histone cluster 1, H2ac	−5.58621	0.01429

**Table 3:** Differentially expressed genes in the hippocampus.

**Gene ID**	**Gene symbol**	**Description**	**Fold change (deaf/control)**	**Raw *p*-value (deaf/control)**
**a. Up-regulated genes**				
14067	F5	Coagulation factor V	11.32650	0.00051
100040591	Kcnj13	Potassium inwardly-rectifying channel, subfamily J, member 13	8.65329	0.01092
11826	Aqp1	Aquaporin 1	7.30121	0.01005
19116	Prlr	Prolactin receptor	6.15455	0.00349
12837	Col8a1	Collagen, type VIII, alpha 1	5.81586	0.00660
73608	Marveld3	MARVEL (membrane-associating) domain containing 3	4.63891	0.00105
96875	Prg4	Proteoglycan 4 (megakaryocyte stimulating factor, articular superficial zone protein)	4.44379	0.00000
434223	Gm1966	Predicted gene 1966	4.08558	0.00000
63873	Trpv4	Transient receptor potential cation channel, subfamily V, member 4	4.08263	0.02017
54612	Sfrp5	Secreted frizzled-related sequence protein 5	4.06183	0.02991
16668	Krt18	Keratin 18	3.99511	0.01639
338403	Cndp1	Carnosine dipeptidase 1 (metallopeptidase M20 family)	3.92104	0.00727
12374	Casr	Calcium-sensing receptor	3.91579	0.00079
14960	H2-Aa	Histocompatibility 2, class II antigen A, alpha	3.75704	0.00265
100169864	Gm44504	Predicted readthrough transcript (NMD candidate), 44504	3.62006	0.04651
12828	Col4a3	Collagen, type IV, alpha 3	3.55651	0.01753
245945	Rbm47	RNA binding motif protein 47	3.55321	0.03480
18606	Enpp2	Ectonucleotide pyrophosphatase/phosphodiesterase 2	3.42590	0.01823
18784	Pla2g5	Phospholipase A2, group V	3.31853	0.03922
16149	Cd74	CD74	3.22550	0.01522
71355	Col24a1	Collagen, type XXIV, alpha 1	3.17166	0.00002
329941	Col8a2	Collagen, type VIII, alpha 2	3.17105	0.02224
246048	Chodl	Chondrolectin	3.08834	0.04821
18542	Pcolce	Procollagen C-endopeptidase enhancer protein	3.05446	0.00968
12737	Cldn1	Claudin 1	3.00102	0.02733
20347	Sema3b	Sema domain, immunoglobulin domain (Ig), short basic domain, secreted, (semaphorin) 3B	2.98934	0.01241
102639145	LOC102639145	Transcription factor SKN7	2.97922	0.00561
208890	Slc26a7	Solute carrier family 26, member 7	2.97441	0.00013
330830	Drc7	Dynein regulatory complex subunit 7	2.86807	0.03430
14089	Fap	Fibroblast activation protein	2.85711	0.02732
100038882	Isg15	ISG15 ubiquitin-like modifier	2.85010	0.00456
16591	Kl	Klotho	2.82507	0.02258
268970	Arhgap28	Rho GTPase activating protein 28	2.81550	0.00376
277328	Trpa1	Transient receptor potential cation channel, subfamily A, member 1	2.79628	0.00108
74732	Stx11	Syntaxin 11	2.76440	0.00776
18400	Slc22a18	Solute carrier family 22 (organic cation transporter), member 18	2.74952	0.00940
217169	Tns4	Tensin 4	2.74538	0.04620
625286	Tmem236	Transmembrane protein 236	2.74531	0.01272
16847	Lepr	Leptin receptor	2.63457	0.02200
16159	Il12a	Interleukin 12a	2.60059	0.00039
243755	Slc13a4	Solute carrier family 13 (sodium/sulfate symporters), member 4	2.58458	0.03987
211577	Mrgprf	MAS-related GPR, member F	2.55384	0.00442
71889	Epn3	Epsin 3	2.54936	0.03085
208943	Myo5c	Myosin VC	2.52633	0.00544
56072	Lgals12	Lectin, galactose binding, soluble 12	2.52565	0.01337
270097	Vat1l	Vesicle amine transport protein 1 like	2.52434	0.00503
74071	Lmntd1	Lamin tail domain containing 1	2.51746	0.00012
217430	Pqlc3	PQ loop repeat containing	2.49585	0.00191
19682	Rdh5	Retinol dehydrogenase 5	2.47215	0.01969
57890	Il17re	Interleukin 17 receptor E	2.44971	0.04852
226691	Ifi207	Interferon activated gene 207	2.44593	0.00009
192212	Prom2	Prominin 2	2.42215	0.00144
66898	Baiap2l1	BAI1-associated protein 2-like 1	2.41073	0.01571
244416	Ppp1r3b	Protein phosphatase 1, regulatory subunit 3B	2.40559	0.00342
353169	Slc2a12	Solute carrier family 2 (facilitated glucose transporter), member 12	2.39779	0.01277
235631	Prss50	Protease, serine 50	2.36875	0.00626
105246303	Gm41607	Predicted gene, 41607	2.35532	0.00008
269823	Pon3	Paraoxonase 3	2.35186	0.00767
242509	Bnc2	Basonuclin 2	2.34916	0.00009
64378	Gpr88	G-protein coupled receptor 88	2.33412	0.00001
94352	Loxl2	Lysyl oxidase-like 2	2.33005	0.01761
277353	Tcfl5	Transcription factor-like 5 (basic helix-loop-helix)	2.32594	0.02125
22626	Slc23a3	Solute carrier family 23 (nucleobase transporters), member 3	2.32299	0.00865
24110	Usp18	Ubiquitin specific peptidase 18	2.29958	0.02345
12841	Col9a3	Collagen, type IX, alpha 3	2.29593	0.00937
102631705	Gm29975	Predicted gene, 29975, transcript variant X1	2.27515	0.00175
20698	Sphk1	Sphingosine kinase 1	2.27148	0.00111
21956	Tnnt2	Troponin T2, cardiac	2.26911	0.00001
11768	Ap1m2	Adaptor protein complex AP-1, mu 2 subunit	2.26132	0.01163
319239	Npsr1	Neuropeptide S receptor 1	2.24119	0.01661
20271	Scn5a	Sodium channel, voltage-gated, type V, alpha	2.20943	0.01370
269120	Optc	Opticin	2.19504	0.01620
243634	Ano2	Anoctamin 2	2.18771	0.00009
667803	H2-T-ps	Histocompatibility 2, T region locus, pseudogene	2.18066	0.02451
23962	Oasl2	2′-5′ oligoadenylate synthetase-like 2	2.17714	0.01093
15957	Ifit1	Interferon-induced protein with tetratricopeptide repeats 1	2.17630	0.01729
217325	Llgl2	LLGL2 scribble cell polarity complex component	2.17085	0.00156
54123	Irf7	Interferon regulatory factor 7	2.16945	0.02250
64058	Perp	PERP, TP53 apoptosis effector	2.16029	0.02229
11856	Arhgap6	Rho GTPase activating protein 6	2.15633	0.00255
12577	Cdkn1c	Cyclin-dependent kinase inhibitor 1C (P57)	2.15592	0.01175
67473	Slc47a1	Solute carrier family 47, member 1	2.15002	0.00787
64242	Ngb	Neuroglobin	2.13014	0.04284
237759	Col23a1	Collagen, type XXIII, alpha 1	2.12340	0.01515
15959	Ifit3	Interferon-induced protein with tetratricopeptide repeats 3	2.12169	0.00910
27375	Tjp3	Tight junction protein 3	2.09354	0.01509
105247220	Gm42355	Predicted gene, 42355, transcript variant X29	2.09040	0.00952
73338	Itpripl1	Inositol 1,4,5-triphosphate receptor interacting protein-like 1	2.09034	0.02630
22420	Wnt6	Wingless-type MMTV integration site family, member 6	2.09021	0.00848
12904	Crabp2	Cellular retinoic acid binding protein II	2.08715	0.00121
105734727	Gm27021	Predicted gene, 27021	2.08179	0.04292
74424	Tmc5	Transmembrane channel-like gene family 5	2.07842	0.02560
14264	Fmod	Fibromodulin	2.07183	0.00313
234582	Ccdc102a	Coiled-coil domain containing 102A	2.06606	0.01392
69550	Bst2	Bone marrow stromal cell antigen 2	2.05917	0.00037
667370	Ifit3b	Interferon-induced protein with tetratricopeptide repeats 3B	2.02357	0.01225
20289	Scx	Scleraxis	2.01667	0.01186
6543	Mdfic	MyoD family inhibitor domain containing	2.01167	0.01726
12835	Col6a3	Collagen, type VI, alpha 3	2.00305	0.00554
**b. Down-regulated genes**				
217216	BC030867	cDNA sequence BC030867	−2.11057	0.00374
211135	D130040H23Rik	RIKEN cDNA D130040H23 gene	−2.11174	0.01995
18071	Nhlh1	Nescient helix loop helix 1	−2.14504	0.00259
240879	Mettl11b	Methyltransferase like 11B	−2.19592	0.02249
12939	Pcdha7	Protocadherin alpha 7	−2.21782	0.04278
15442	Hpse	Heparanase	−2.25788	0.00477
74589	Kbtbd12	Kelch repeat and BTB (POZ) domain containing 12	−2.27407	0.03156
210853	Zfp947	Zinc finger protein 947	−2.28188	0.00010
116903	Calcb	Calcitonin-related polypeptide, beta	−2.31538	0.02406
320604	Ccdc169	Coiled-coil domain containing 169	−2.37904	0.02867
69852	Tcf23	Transcription factor 23	−2.44860	0.00361
75015	4930503B20Rik	RIKEN cDNA 4930503B20 gene	−2.44869	0.02793
97122	H4c14	Histone cluster 2, H4	−2.50098	0.02006
383592	Kif28	Kinesin family member 28	−2.90396	0.01641
115488671	LOC115488671	Uncharacterized LOC115488671	−3.20361	0.02140

### Results of GO analysis

#### Cochlea

**Figure 3:**
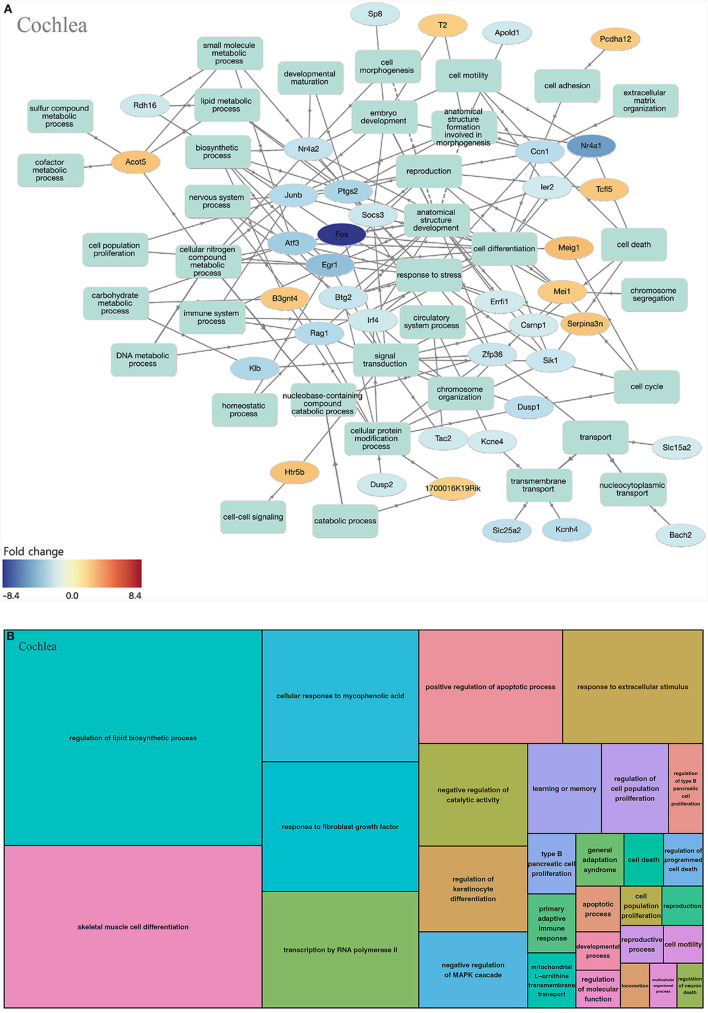
GO analysis on biological process of the cochlea. **(A)** Term annotation analysis from GOnet. Many genes are turned out down-regulated. **(B)** Treemap analysis from REVIGO. Many GOs can be categorized into cell proliferation and apoptosis.

When we analyzed the cochlea, 294 GOs were identified as statistically significant (biological process, 259; molecular function, 34; cellular component, 1). The highly enriched GOs in the biological processes of the cochlea were mostly networked with down-regulated genes (Figure 3A). When the GOs of the biological process were classified into upper categories by the treemap technique, many categories were related to cell proliferation (regulation of lipid biosynthetic process, skeletal muscle cell differentiation, transcription by RNA polymerase II, response to fibroblast growth factor, and regulation of keratinocyte differentiation). There were other categories related to apoptosis [positive regulation of the apoptotic process, negative regulation of the mitogen-activated protein kinase (MAPK) cascade, regulation of cell death, and cell death; Figure 3B, Supplementary Table 1].

#### Auditory cortex

**Figure 4:**
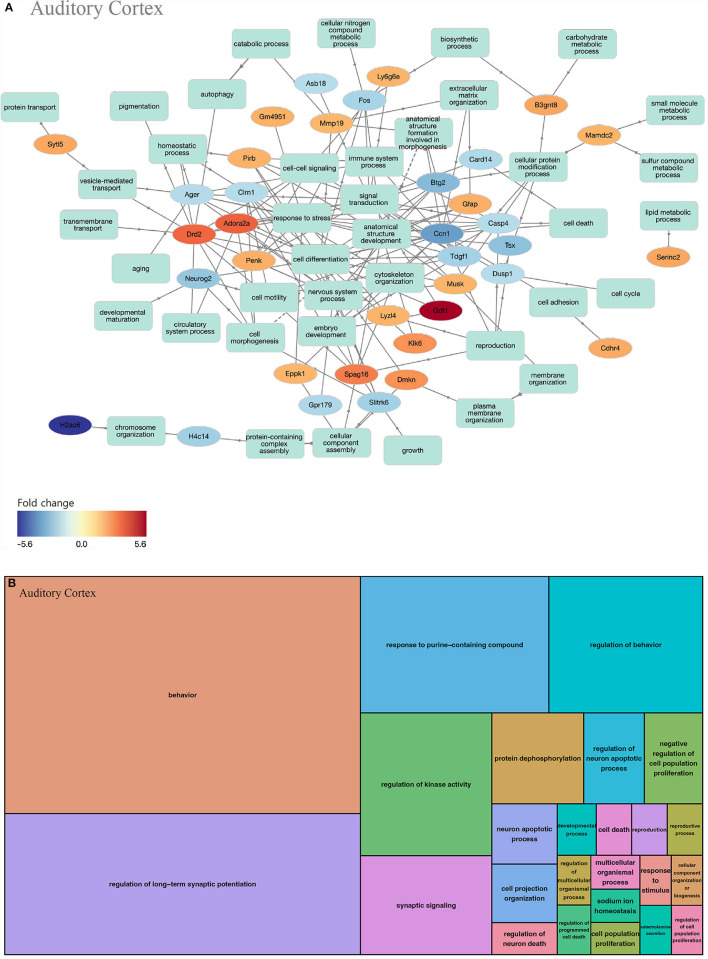
GO analysis on biological process of the auditory cortex. **(A)** Term annotation analysis from GOnet. Up-regulated genes and down-regulated genes are mixed to a similar degree. **(B)** Treemap analysis from REVIGO. GOs can be classified into behavior-related, synapse-related, cell signaling, cell metabolism, and apoptosis categories.

In the auditory cortex, 211 GOs were identified (biological processes, 193; molecular functions, 1; and cell components, 17). In the biological processes of the auditory cortex, the up-regulated and down-regulated genes play similar roles (Figure 4A). GOs of the biological process can be classified as behavior-concerning (behavior and regulation of behavior), synapse-related (regulation of long-term synaptic potentiation and synaptic signaling), cell-signaling (response to purine-containing compound and regulation of kinase activity), cell metabolism (response to purine-containing compound, regulation of kinase activity, and protein dephosphorylation), and apoptosis (regulation of neuron death) categories, among others (Figure 4B, Supplementary Table 2).

#### Hippocampus

**Figure 5:**
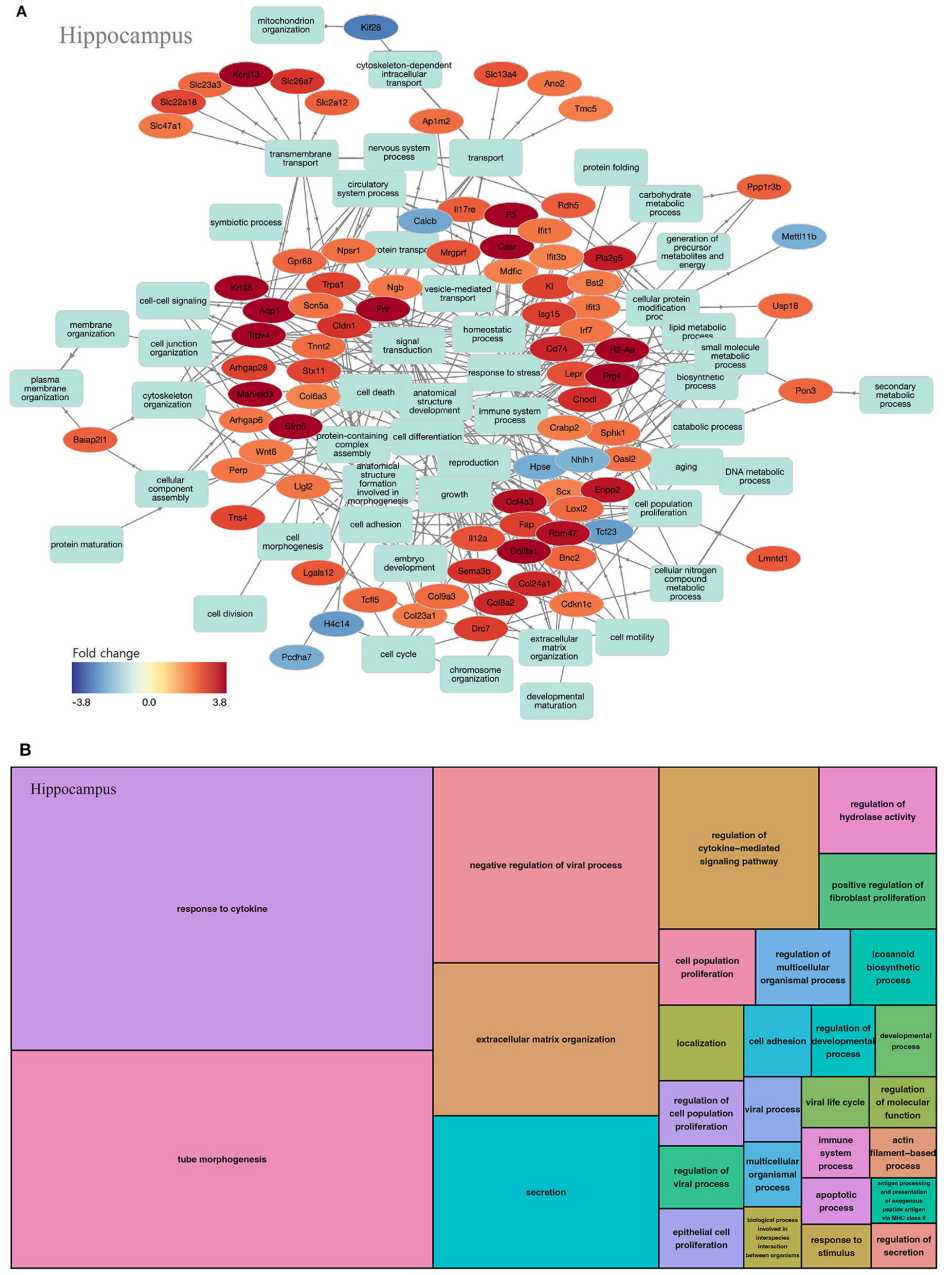
GO analysis on biological process of the hippocampus. **(A)** Term annotation analysis from GOnet. Most genes are up-regulated. **(B)** Treemap analysis from REVIGO. GOs can be classified into inflammation-related and viral infection-related categories.

In the hippocampus, 203 GOs were identified (biological processes, 137; molecular functions, 40; and cell components, 26). In the biological process of the hippocampus, most key genes were upregulated (Figure 5A). GOs of the biological process can be classified as inflammation-related (response to cytokine, regulation of cytokine-mediated signaling pathway, and positive regulation of fibroblast proliferation) and viral infection-related categories (negative regulation of the viral process, regulation of viral process, extracellular matrix organization, secretion, and regulation of hydrolase activity, among others; Figure 5B, Supplementary Table 3).

### KEGG pathway results

#### Cochlea

Ten KEGG pathways were statistically significant. These included pathways related to immune responses, such as the tumor necrosis factor (TNF) signaling pathway, and those related to apoptosis, such as the MAPK signaling pathway. Twelve genes were included in these pathways, most of which were down-regulated (Table 4).

**Table 4:** Statistically significant KEGG pathways in the cochlea.

**Map ID**	**Map name**	***p*-value**	**Gene ID**	**Gene symbol**	**Description**	**Fold change (deaf/control)**	**Raw *p*-value (deaf/control)**
04668	TNF signaling pathway	< 0.001	12702	Socs3	Suppressor of cytokine signaling 3	−2.241	0.005
			14281	Fos	FBJ osteosarcoma oncogene	−8.354	< 0.001
			16477	Junb	Jun B proto-oncogene	−3.012	< 0.001
			19225	Ptgs2	Prostaglandin-endoperoxide synthase 2	−3.255	0.004
04010	MAPK signaling pathway	0.001	13537	Dusp2	Dual specificity phosphatase 2	−2.121	< 0.001
			14281	Fos	FBJ osteosarcoma oncogene	−8.354	< 0.001
			15370	Nr4a1	Nuclear receptor subfamily 4, group A, member 1	−5.280	< 0.001
			19252	Dusp1	Dual specificity phosphatase 1	−2.804	< 0.001
04935	Growth hormone synthesis, secretion and action	0.003	12702	Socs3	Suppressor of cytokine signaling 3	−2.241	0.005
			14281	Fos	FBJ osteosarcoma oncogene	−8.354	< 0.001
			16477	Junb	Jun B proto-oncogene	−3.012	< 0.001
04380	Osteoclast differentiation	0.004	12702	Socs3	Suppressor of cytokine signaling 3	−2.241	0.005
			14281	Fos	FBJ osteosarcoma oncogene	−8.354	< 0.001
			16477	Junb	Jun B proto-oncogene	−3.012	< 0.001
04928	Parathyroid hormone synthesis, secretion and action	0.003	13653	Egr1	Early growth response 1	−4.087	0.002
			14281	Fos	FBJ osteosarcoma oncogene	−8.354	< 0.001
			18227	Nr4a2	Nuclear receptor subfamily 4, group A, member 2	−2.527	0.001
04726	Serotonergic synapse	0.004	15564	Htr5b	5-hydroxytryptamine (serotonin) receptor 5B	2.557	0.007
			19225	Ptgs2	Prostaglandin-endoperoxide synthase 2	−3.255	0.004
			19252	Dusp1	Dual specificity phosphatase 1	−2.804	< 0.001
05167	Kaposi sarcoma-associated herpesvirus infection	0.011	14281	Fos	FBJ osteosarcoma oncogene	−8.354	< 0.001
			19225	Ptgs2	Prostaglandin-endoperoxide synthase 2	−3.255	0.004
			22695	Zfp36	Zinc finger protein 36	−2.450	0.006
05166	Human T-cell leukemia virus 1 infection	0.014	13653	Egr1	Early growth response 1	−4.087	0.002
			14281	Fos	FBJ osteosarcoma oncogene	−8.354	< 0.001
			22695	Zfp36	Zinc finger protein 36	−2.450	0.006
05140	Leishmaniasis	0.050	14281	Fos	FBJ osteosarcoma oncogene	−8.354	< 0.001
			19225	Ptgs2	Prostaglandin-endoperoxide synthase 2	−3.255	0.004
04913	Ovarian steroidogenesis	0.045	19225	Ptgs2	Prostaglandin-endoperoxide synthase 2	−3.255	0.004
			217698	Acot5	Acyl-CoA thioesterase 5	2.532	0.003

#### Auditory cortex

Seven KEGG pathways were statistically significant. Pathways related to immune reactions (neutrophil extracellular trap formation and B cell receptor signaling pathway), cell death [cyclic adenosine monophosphate (cAMP) signaling pathway], and neuronal synapses (neuroactive ligand-receptor interaction) were expressed. Eleven genes were found in these pathways, and the up-regulated and downregulated genes were combined (Table 5).

**Table 5:** Statistically significant KEGG pathways in the auditory cortex.

**Map ID**	**Map name**	***p*-value**	**Gene ID**	**Gene symbol**	**Description**	**Fold change (deaf/control)**	**Raw *p*-value (deaf/control)**
05034	Alcoholism	< 0.001	11540	Adora2a	Adenosine A2a receptor	3.562	0.001
			13489	Drd2	Dopamine receptor D2	3.514	0.004
			97122	H4c14	Histone cluster 2, H4	−2.434	0.026
			319164	H2ac6	Histone cluster 1, H2ac	−5.586	0.014
			665622	Hist1h2br	HISTONE cluster 1 H2br	−2.001	0.031
04613	Neutrophil extracellular trap formation	< 0.001	11596	Ager	Advanced glycosylation end product-specific receptor	−2.053	0.004
			12363	Casp4	Caspase 4, apoptosis-related cysteine peptidase	−2.043	0.013
			97122	H4c14	Histone cluster 2, H4	−2.434	0.026
			319164	H2ac6	Histone cluster 1, H2ac	−5.586	0.014
			665622	Hist1h2br	Histone cluster 1 H2br	−2.001	0.031
05322	Systemic lupus erythematosus	0.003	97122	H4c14	Histone cluster 2, H4	−2.434	0.026
			319164	H2ac6	Histone cluster 1, H2ac	−5.586	0.014
			665622	Hist1h2br	Histone cluster 1 H2br	−2.001	0.031
04024	cAMP signaling pathway	0.006	11540	Adora2a	Adenosine A2a receptor	3.562	0.001
			13489	Drd2	Dopamine receptor D2	3.514	0.004
			14281	Fos	FBJ osteosarcoma oncogene	−2.302	0.044
05012	Parkinson's disease	0.009	11540	Adora2a	Adenosine A2a receptor	3.562	0.001
			13489	Drd2	Dopamine receptor D2	3.514	0.004
			19252	Dusp1	Dual specificity phosphatase 1	−2.048	0.001
04080	Neuroactive ligand-receptor interaction	0.017	11540	Adora2a	Adenosine A2a receptor	3.562	0.001
			13489	Drd2	Dopamine receptor D2	3.514	0.004
			18619	Penk	Preproenkephalin	2.136	0.011
04662	B cell receptor signaling pathway	0.043	14281	Fos	FBJ osteosarcoma oncogene	−2.302	0.044
			18733	Pirb	Paired Ig-like receptor B	2.099	0.003

#### Hippocampus

Seventeen KEGG pathways were statistically significant. These include inflammatory pathways [cytokine-cytokine receptor interaction and the Janus kinases-signal transducer and activator of transcription proteins (JAK-STAT) signaling pathway] and viral infection-related pathways. Twenty-seven genes were involved in these pathways, most of which were up-regulated (Table 6).

**Table 6:** Statistically significant KEGG pathways in the hippocampus.

**Map ID**	**Map name**	***p*-value**	**Gene ID**	**Gene Symbol**	**Description**	**Fold change (deaf/control)**	**Raw *p*-value (deaf/control)**
04974	Protein digestion and absorption	< 0.001	12828	Col4a3	Collagen, type IV, alpha 3	3.557	0.018
			12835	Col6a3	Collagen, type VI, alpha 3	2.003	0.006
			12837	Col8a1	Collagen, type VIII, alpha 1	5.816	0.007
			12841	Col9a3	Collagen, type IX, alpha 3	2.296	0.009
			71355	Col24a1	Collagen, type XXIV, alpha 1	3.172	0.000
			237759	Col23a1	Collagen, type XXIII, alpha 1	2.123	0.015
			329941	Col8a2	Collagen, type VIII, alpha 2	3.171	0.022
			100040591	Kcnj13	Potassium inwardly-rectifying channel, subfamily J, member 13	8.653	0.011
05165	Human papillomavirus infection	< 0.001	12828	Col4a3	Collagen, type IV, alpha 3	3.557	0.018
			12835	Col6a3	Collagen, type VI, alpha 3	2.003	0.006
			12841	Col9a3	Collagen, type IX, alpha 3	2.296	0.009
			22420	Wnt6	Wingless-type MMTV integration site family, member 6	2.090	0.008
			23962	Oasl2	2'-5' oligoadenylate synthetase-like 2	2.177	0.011
			217325	Llgl2	LLGL2 scribble cell polarity complex component	2.171	0.002
			667803	H2-T-ps	Histocompatibility 2, T region locus, pseudogene	2.181	0.025
			100038882	Isg15	ISG15 ubiquitin-like modifier	2.850	0.005
05168	Herpes simplex virus 1 infection	< 0.001	14960	H2-Aa	Histocompatibility 2, class II antigen A, alpha	3.757	0.003
			16149	Cd74	CD74 antigen	3.225	0.015
			16159	Il12a	Interleukin 12a	2.601	0.000
			54123	Irf7	Interferon regulatory factor 7	2.169	0.023
			69550	Bst2	Bone marrow stromal cell antigen 2	2.059	0.000
			210853	Zfp947	Zinc finger protein 947	−2.282	0.000
			667803	H2-T-ps	Histocompatibility 2, T region locus, pseudogene	2.181	0.025
04530	Tight junction	0.005	12737	Cldn1	Claudin 1	3.001	0.027
			27375	Tjp3	Tight junction protein 3	2.094	0.015
			73608	Marveld3	MARVEL domain containing 3	4.639	0.001
			217325	Llgl2	LLGL2 scribble cell polarity complex component	2.171	0.002
05152	Tuberculosis	0.006	14960	H2-Aa	Histocompatibility 2, class II antigen A, alpha	3.757	0.003
			16149	Cd74	CD74 antigen	3.225	0.015
			16159	Il12a	Interleukin 12a	2.601	< 0.001
			20698	Sphk1	Sphingosine kinase 1	2.271	0.001
05330	Allograft rejection	0.007	14960	H2-Aa	Histocompatibility 2, class II antigen A, alpha	3.757	0.003
			16159	Il12a	Interleukin 12a	2.601	< 0.001
			667803	H2-T-ps	Histocompatibility 2, T region locus, pseudogene	2.181	0.025
04622	RIG-I-like receptor signaling pathway	0.009	16159	Il12a	Interleukin 12a	2.601	< 0.001
			54123	Irf7	Interferon regulatory factor 7	2.169	0.023
			100038882	Isg15	ISG15 ubiquitin-like modifier	2.850	0.005
04940	Type I diabetes mellitus	0.009	14960	H2-Aa	Histocompatibility 2, class II antigen A, alpha	3.757	0.003
			16159	Il12a	Interleukin 12a	2.601	< 0.001
			667803	H2-T-ps	Histocompatibility 2, T region locus, pseudogene	2.181	0.025
05169	Epstein-Barr virus infection	0.012	14960	H2-Aa	Histocompatibility 2, class II antigen A, alpha	3.757	0.003
			54123	Irf7	Interferon regulatory factor 7	2.169	0.023
			667803	H2-T-ps	Histocompatibility 2, T region locus, pseudogene	2.181	0.025
			100038882	Isg15	ISG15 ubiquitin-like modifier	2.850	0.005
04512	ECM-receptor interaction	0.014	12828	Col4a3	Collagen, type IV, alpha 3	3.557	0.018
			12835	Col6a3	Collagen, type VI, alpha 3	2.003	0.006
			12841	Col9a3	Collagen, type IX, alpha 3	2.296	0.009
04612	Antigen processing and presentation	0.015	14960	H2-Aa	Histocompatibility 2, class II antigen A, alpha	3.757	0.003
			16149	Cd74	CD74 antigen	3.225	0.015
			667803	H2-T-ps	Histocompatibility 2, T region locus, pseudogene	2.181	0.025
04060	Cytokine-cytokine receptor interaction	0.022	16159	Il12a	Interleukin 12a	2.601	< 0.001
			16847	Lepr	Leptin receptor	2.635	0.022
			19116	Prlr	prolactin receptor	6.155	0.003
			57890	Il17re	Interleukin 17 receptor E	2.450	0.049
04151	PI3K-Akt signaling pathway	0.037	12828	Col4a3	Collagen, type IV, alpha 3	3.557	0.018
			12835	Col6a3	Collagen, type VI, alpha 3	2.003	0.006
			12841	Col9a3	Collagen, type IX, alpha 3	2.296	0.009
			19116	Prlr	Prolactin receptor	6.155	0.003
05160	Hepatitis C	0.045	12737	Cldn1	Claudin 1	3.001	0.027
			15957	Ifit1	Interferon-induced protein with tetratricopeptide repeats 1	2.176	0.017
			54123	Irf7	Interferon regulatory factor 7	2.169	0.023
04630	JAK-STAT signaling pathway	0.046	16159	Il12a	Interleukin 12a	2.601	< 0.001
			16847	Lepr	Leptin receptor	2.635	0.022
			19116	Prlr	Prolactin receptor	6.155	0.003
05164	Influenza A	0.049	14960	H2-Aa	Histocompatibility 2, class II antigen A, alpha	3.757	0.003
			16159	Il12a	Interleukin 12a	2.601	< 0.001
			54123	Irf7	Interferon regulatory factor 7	2.169	0.023
04514	Cell adhesion molecules	0.049	12737	Cldn1	Claudin 1	3.001	0.027
			14960	H2-Aa	Histocompatibility 2, class II antigen A, alpha	3.757	0.003
			667803	H2-T-ps	Histocompatibility 2, T region locus, pseudogene	2.181	0.025

## Discussion

We conducted an omics study to determine how hearing loss affects the central auditory pathway, including cognitive organs. We examined the effects of hearing loss on the cochlea, auditory cortex, and hippocampus using mRNA sequencing. First, we compared the DEGs in these organs. The number of up-regulated genes was the lowest in the cochlea, and it increased toward the central nervous system. In contrast, the number of down-regulated genes increased as we moved toward the periphery (Tables 1–3, Figures 3A, 4A, 5A). Most DEGs in the cochlea exhibited an inhibitory pattern, whereas those in the hippocampus were excitatory.

In the cochlea, genes related to activator protein 1 (AP-1) transcription (*Fos* and *Junb*) and inflammation (*Ptgs2* and *Socs3*) were down-regulated. AP-1 transcription factors control cell differentiation, proliferation, and apoptosis during stress and infections (Ameyar et al., 2003; Hess et al., 2004). This suggests that the peak time of inflammatory response and cell death has passed. In the auditory cortex, genes related to the guanine nucleotide-binding protein (G protein)-coupled receptor superfamily (*Drd2* and *Adora2a*) were up-regulated. They are known to have relation with regulation of cognitive function and mood by promoting dopamine binding and dopamine neurotransmitter receptor activity (Komatsu et al., 2014; Khlghatyan et al., 2019). In the hippocampus, genes related to the major histocompatibility complex (*H2-T-ps, H2-Aa, Cd74*), helper T cell type 1 (*Il12a*), and the extracellular matrix (*Col4a3, Col6a3, Col9a3*) were up-regulated, implying an actively processed adaptive immune reactions and cellular changes.

When examining the GOs and KEGG pathways, we observed that the expression patterns differed for each organ. Genes related to cell proliferation and death were simultaneously expressed in the cochlea (Figure 3B, Supplementary Table 1). The TNF signaling pathway, associated with the immune response, and the MAPK signaling pathway, related to apoptosis, were also activated (Table 4). However, most gene activities were down-regulated. Considering cell proliferation, inflammation, and apoptosis occur simultaneously after hearing loss in the cochlea (Shu et al., 2019; Milon et al., 2021; Warnecke et al., 2021; Chen et al., 2022; Paciello et al., 2023), all of these processes seem to be in the finishing stage.

In the auditory cortex, the GO patterns were quite different. Genes related to behavior and neuronal synapse function were highly expressed, and those associated with cellular metabolism, cell signaling, and apoptosis were also observed (Figure 4B, Supplementary Table 2). The cAMP signaling pathway and neuroactive ligand-receptor interactions seem to be related to neuronal cell death and synaptic activity (Table 5). Therefore, it can be inferred that brain plasticity mechanisms occur through synaptic changes along with neuronal cell death, affecting cognitive function in the auditory cortex. Notably, alcoholism and Parkinson's disease appeared in the KEGG pathway, suggesting that changes in the auditory cortex following hearing loss progress in a similar pattern to these disorders.

The hippocampus differs from the other two organs in that most genes exhibited excitatory activity (Figure 5A). Inflammation-related pathways, such as cytokine-cytokine receptor interactions, were commonly activated, indicating ongoing inflammation (Figure 5B, Table 6, Supplementary Table 3). Interestingly, there are many pathways related to viral and fungal infections that seem unrelated, suggesting that when the hippocampus is challenged, it exhibits a response similar to infection. Thus, neuroinflammation persists in the hippocampus even after 3 months of hearing loss.

**Figure 6:**
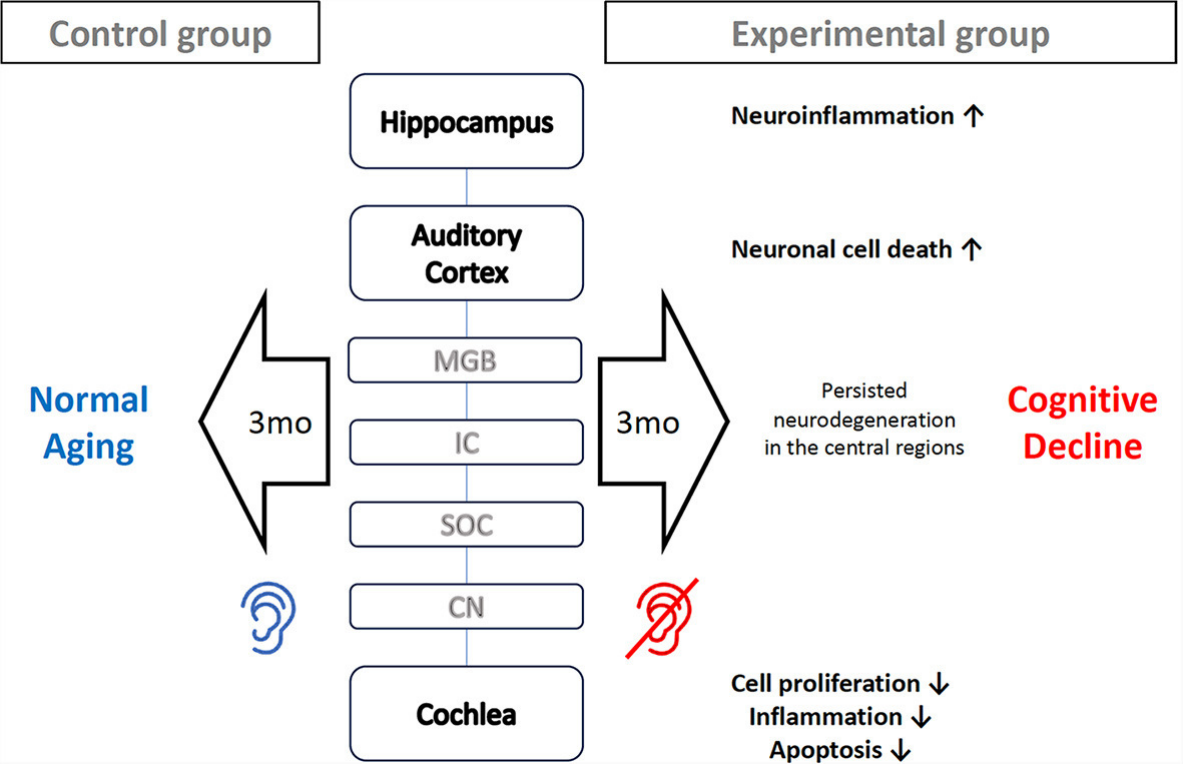
Proposed mechanism how noise-induced hearing loss cause mice cognitive decline. Three months after the onset of noise-induced hearing loss, processes such as cell proliferation, inflammation, and apoptosis come to finishing stage in the cochlea. However, neuronal cell death is still progressing in the auditory cortex, and neuroinflammation persists in the hippocampus. These prolonged neurodegeneration processes in the central region accelerates cognitive decline.

In conclusion, apoptosis and inflammation persisted more actively in the order of hippocampus, auditory cortex, cochlea in the long term (12 weeks) after noise-induced hearing loss. This implies that the neurodegenerative effects of noise exposure do not resolve quickly in the central regions but persist for a considerable period. Therefore, cognitive decline following noise-induced hearing loss is likely to be a progressive process rather than an instant deterioration (Figure 6). Some studies see the cognitive decline not just as elongated degeneration, but as accelerated aging (Zhuang et al., 2020; Paciello et al., 2021). In the case of normal C57BL/6J mice, no histological changes are observed in the auditory cortex or hippocampus up to 6 months of age (Dong et al., 2018). However, it appears that there are gradual changes in proteins that play significant roles in plasticity and cognitive functions, such as MMP-9 (Dong et al., 2018). Hence, proactive treatment to prevent the progression of cognitive decline remain of substantial importance even if there are little chance of auditory restoration. For example, auditory rehabilitation strategies, including the utilization of hearing aids or cochlear implants, as well as medical interventions such as antioxidant therapy, may prove beneficial for patients with hearing impairment in mitigating the risk of cognitive decline.

This study has a few limitations. First, this study was conducted only in the aspect of mRNA expression, so it may appear differently at the level of protein. Secondly, this was a cross-sectional study that included 24-week-old mice, and we analyzed its results by considering the general progression of neuroinflammation. However, a time-series study of each organ is required to determine the dynamic mechanisms of neuroinflammation accurately. Finally, the noise used in this study was large enough to evoked permanent hearing loss in a single exposure. Loud noise can affect the central auditory pathway and the hippocampus within a few days (Groschel et al., 2018; Chen et al., 2022). And long-term exposure to noise could affect central neural system differently. In particular, the effects on cognitive function may differ significantly in cases of noise stress or gradual hearing deterioration due to sustained high-intensity noise. Further studies are required to generalize these findings to the overall context of noise-induced hearing loss.

## Data availability statement

The datasets presented in this study can be found in online repositories. The names of the repository/repositories and accession number(s) can be found at: https://www.ncbi.nlm.nih.gov/bioproject/PRJNA1061000/.

## Ethics statement

The animal study was approved by Institutional Animal Care and Use Committee (IACUC) of Boramae Hospital (IACUC number 2022-0133). The study was conducted in accordance with the local legislation and institutional requirements.

## Author contributions

S-YL: Data curation, Formal analysis, Investigation, Software, Visualization, Writing—original draft. HL: Data curation, Investigation, Methodology, Validation, Writing—review & editing. M-HP: Conceptualization, Funding acquisition, Investigation, Methodology, Project administration, Supervision, Writing—review & editing.
