# Supplementary material for: Transcriptomic analysis reveals prolonged neurodegeneration in the hippocampus of adult C57BL/6N mouse deafened by noise
Source: Front Neurosci. 2024 Feb 12;18:1340854. doi: 10.3389/fnins.2024.1340854 (PMC10894918; doi:10.3389/fnins.2024.1340854)
Supplement: Supplementary file 1 [file Table_1.DOCX]

# Supplementary Tables

Supplementary Table 1. Specified GOs in each category of the cochlea.

| Category | Gene Ontology ID | Gene Ontology Description |
| --- | --- | --- |
| regulation of lipid biosynthetic process | GO:0046890 | regulation of lipid biosynthetic process |
|  | GO:0000122 | negative regulation of transcription by RNA polymerase II |
|  | GO:0006357 | regulation of transcription by RNA polymerase II |
|  | GO:0009889 | regulation of biosynthetic process |
|  | GO:0009890 | negative regulation of biosynthetic process |
|  | GO:0009891 | positive regulation of biosynthetic process |
|  | GO:0009892 | negative regulation of metabolic process |
|  | GO:0010468 | regulation of gene expression |
|  | GO:0010565 | regulation of cellular ketone metabolic process |
|  | GO:0010648 | negative regulation of cell communication |
|  | GO:0010868 | negative regulation of triglyceride biosynthetic process |
|  | GO:0019216 | regulation of lipid metabolic process |
|  | GO:0019219 | regulation of nucleobase-containing compound metabolic process |
|  | GO:0023057 | negative regulation of signaling |
|  | GO:0030162 | regulation of proteolysis |
|  | GO:0031323 | regulation of cellular metabolic process |
|  | GO:0031325 | positive regulation of cellular metabolic process |
|  | GO:0031326 | regulation of cellular biosynthetic process |
|  | GO:0043618 | regulation of transcription from RNA polymerase II promoter in response to stress |
|  | GO:0043620 | regulation of DNA-templated transcription in response to stress |
|  | GO:0045935 | positive regulation of nucleobase-containing compound metabolic process |
|  | GO:0048523 | negative regulation of cellular process |
|  | GO:0048585 | negative regulation of response to stimulus |
|  | GO:0050732 | negative regulation of peptidyl-tyrosine phosphorylation |
|  | GO:0051171 | regulation of nitrogen compound metabolic process |
|  | GO:0051172 | negative regulation of nitrogen compound metabolic process |
|  | GO:0051246 | regulation of protein metabolic process |
|  | GO:0051248 | negative regulation of protein metabolic process |
|  | GO:0051252 | regulation of RNA metabolic process |
|  | GO:0051254 | positive regulation of RNA metabolic process |
|  | GO:0060255 | regulation of macromolecule metabolic process |
|  | GO:0062012 | regulation of small molecule metabolic process |
|  | GO:0062197 | cellular response to chemical stress |
|  | GO:0080090 | regulation of primary metabolic process |
|  | GO:1901834 | regulation of deadenylation-independent decapping of nuclear-transcribed mRNA |
| skeletal muscle cell differentiation | GO:0035914 | skeletal muscle cell differentiation |
|  | GO:0001706 | endoderm formation |
|  | GO:0001890 | placenta development |
|  | GO:0001944 | vasculature development |
|  | GO:0002041 | intussusceptive angiogenesis |
|  | GO:0002042 | cell migration involved in sprouting angiogenesis |
|  | GO:0002331 | pre-B cell allelic exclusion |
|  | GO:0002521 | leukocyte differentiation |
|  | GO:0007275 | multicellular organism development |
|  | GO:0009653 | anatomical structure morphogenesis |
|  | GO:0009888 | tissue development |
|  | GO:0021538 | epithalamus development |
|  | GO:0021986 | habenula development |
|  | GO:0030154 | cell differentiation |
|  | GO:0030216 | keratinocyte differentiation |
|  | GO:0045444 | fat cell differentiation |
|  | GO:0048513 | animal organ development |
|  | GO:0048598 | embryonic morphogenesis |
|  | GO:0048646 | anatomical structure formation involved in morphogenesis |
|  | GO:0048731 | system development |
|  | GO:0048856 | anatomical structure development |
|  | GO:0048869 | cellular developmental process |
|  | GO:0060537 | muscle tissue development |
|  | GO:0060674 | placenta blood vessel development |
|  | GO:0061061 | muscle structure development |
|  | GO:0072223 | metanephric glomerular mesangium development |
|  | GO:0072359 | circulatory system development |
| cellular response to mycophenolic acid | GO:0071506 | cellular response to mycophenolic acid |
|  | GO:0009314 | response to radiation |
|  | GO:0009416 | response to light stimulus |
|  | GO:0010033 | response to organic substance |
|  | GO:0010335 | response to non-ionic osmotic stress |
|  | GO:0014070 | response to organic cyclic compound |
|  | GO:0051602 | response to electrical stimulus |
|  | GO:0070887 | cellular response to chemical stimulus |
|  | GO:0071248 | cellular response to metal ion |
|  | GO:0071470 | cellular response to osmotic stress |
|  | GO:0071471 | cellular response to non-ionic osmotic stress |
|  | GO:0071505 | response to mycophenolic acid |
|  | GO:0098758 | response to interleukin-8 |
|  | GO:0098759 | cellular response to interleukin-8 |
|  | GO:1901698 | response to nitrogen compound |
|  | GO:1901700 | response to oxygen-containing compound |
| transcription by RNA polymerase II | GO:0006366 | transcription by RNA polymerase II |
|  | GO:0006351 | DNA-templated transcription |
|  | GO:0008610 | lipid biosynthetic process |
|  | GO:0009058 | biosynthetic process |
|  | GO:0016598 | protein arginylation |
|  | GO:0018130 | heterocycle biosynthetic process |
|  | GO:0019438 | aromatic compound biosynthetic process |
|  | GO:0032788 | saturated monocarboxylic acid metabolic process |
|  | GO:0032789 | unsaturated monocarboxylic acid metabolic process |
|  | GO:0044283 | small molecule biosynthetic process |
|  | GO:0090360 | platelet-derived growth factor production |
|  | GO:0120254 | olefinic compound metabolic process |
|  | GO:1901362 | organic cyclic compound biosynthetic process |
| response to fibroblast growth factor | GO:0071774 | response to fibroblast growth factor |
|  | GO:0001975 | response to amphetamine |
|  | GO:0009725 | response to hormone |
|  | GO:0014075 | response to amine |
|  | GO:0043434 | response to peptide hormone |
|  | GO:0043435 | response to corticotropin-releasing hormone |
|  | GO:0044344 | cellular response to fibroblast growth factor stimulus |
|  | GO:0070848 | response to growth factor |
|  | GO:0070849 | response to epidermal growth factor |
|  | GO:0071364 | cellular response to epidermal growth factor stimulus |
|  | GO:0071376 | cellular response to corticotropin-releasing hormone stimulus |
|  | GO:1901701 | cellular response to oxygen-containing compound |
| response to extracellular stimulus | GO:0009991 | response to extracellular stimulus |
|  | GO:0000165 | MAPK cascade |
|  | GO:0006950 | response to stress |
|  | GO:0007167 | enzyme-linked receptor protein signaling pathway |
|  | GO:0009605 | response to external stimulus |
|  | GO:0009628 | response to abiotic stimulus |
|  | GO:0009719 | response to endogenous stimulus |
|  | GO:0031668 | cellular response to extracellular stimulus |
|  | GO:0038066 | p38MAPK cascade |
|  | GO:0070371 | ERK1 and ERK2 cascade |
|  | GO:0071496 | cellular response to external stimulus |
| positive regulation of apoptotic process | GO:0043065 | positive regulation of apoptotic process |
|  | GO:0014860 | neurotransmitter secretion involved in regulation of skeletal muscle contraction |
|  | GO:0032350 | regulation of hormone metabolic process |
|  | GO:0032352 | positive regulation of hormone metabolic process |
|  | GO:0043068 | positive regulation of programmed cell death |
|  | GO:0048522 | positive regulation of cellular process |
|  | GO:0060211 | regulation of nuclear-transcribed mRNA poly(A) tail shortening |
|  | GO:0060213 | positive regulation of nuclear-transcribed mRNA poly(A) tail shortening |
|  | GO:1901835 | positive regulation of deadenylation-independent decapping of nuclear-transcribed mRNA |
|  | GO:1903984 | positive regulation of TRAIL-activated apoptotic signaling pathway |
|  | GO:1904582 | positive regulation of intracellular mRNA localization |
| negative regulation of catalytic activity | GO:0043086 | negative regulation of catalytic activity |
|  | GO:0044092 | negative regulation of molecular function |
|  | GO:0050790 | regulation of catalytic activity |
|  | GO:0051338 | regulation of transferase activity |
|  | GO:0052548 | regulation of endopeptidase activity |
|  | GO:1904245 | regulation of polynucleotide adenylyltransferase activity |
|  | GO:1904246 | negative regulation of polynucleotide adenylyltransferase activity |
| regulation of keratinocyte differentiation | GO:0045616 | regulation of keratinocyte differentiation |
|  | GO:0030856 | regulation of epithelial cell differentiation |
|  | GO:0045595 | regulation of cell differentiation |
|  | GO:0045597 | positive regulation of cell differentiation |
|  | GO:0045682 | regulation of epidermis development |
|  | GO:0090361 | regulation of platelet-derived growth factor production |
|  | GO:0090362 | positive regulation of platelet-derived growth factor production |
| negative regulation of MAPK cascade | GO:0043409 | negative regulation of MAPK cascade |
|  | GO:0032227 | negative regulation of synaptic transmission, dopaminergic |
|  | GO:0043408 | regulation of MAPK cascade |
|  | GO:0045786 | negative regulation of cell cycle |
|  | GO:0070373 | negative regulation of ERK1 and ERK2 cascade |
|  | GO:1902532 | negative regulation of intracellular signal transduction |
| learning or memory | GO:0007611 | learning or memory |
|  | GO:0001659 | temperature homeostasis |
|  | GO:0007610 | behavior |
|  | GO:0050890 | cognition |
| regulation of cell population proliferation | GO:0042127 | regulation of cell population proliferation |
|  | GO:0019222 | regulation of metabolic process |
|  | GO:0048519 | negative regulation of biological process |
|  | GO:0051239 | regulation of multicellular organismal process |
| type B pancreatic cell proliferation | GO:0044342 | type B pancreatic cell proliferation |
|  | GO:0033687 | osteoblast proliferation |
| general adaptation syndrome | GO:0051866 | general adaptation syndrome |
|  | GO:0033554 | cellular response to stress |
| regulation of type B pancreatic cell proliferation | GO:0061469 | regulation of type B pancreatic cell proliferation |
|  | GO:0072301 | regulation of metanephric glomerular mesangial cell proliferation |
| primary adaptive immune response | GO:0090720 | primary adaptive immune response |
|  | GO:0090721 | primary adaptive immune response involving T cells and B cells |
| mitochondrial L-ornithine transmembrane transport | GO:1990575 | mitochondrial L-ornithine transmembrane transport |
|  | GO:0051674 | localization of cell |
| - | GO:0000003 | reproduction |
| - | GO:0006915 | apoptotic process |
| - | GO:0008219 | cell death |
| - | GO:0008283 | cell population proliferation |
| - | GO:0022414 | reproductive process |
| - | GO:0032501 | multicellular organismal process |
| - | GO:0032502 | developmental process |
| - | GO:0040011 | locomotion |
| - | GO:0043067 | regulation of programmed cell death |
| - | GO:0048870 | cell motility |
| - | GO:0065009 | regulation of molecular function |
| - | GO:1901214 | regulation of neuron death |

Supplementary Table 2. Specified GOs in each category of the auditory cortex.

| Category | Gene Ontology ID | Gene Ontology Description |
| --- | --- | --- |
| behavior | GO:0097028 | positive regulation of nervous system development |
|  | GO:0060007 | regulation of glial cell-derived neurotrophic factor production |
|  | GO:0051867 | positive regulation of glial cell-derived neurotrophic factor production |
|  | GO:0051866 | regulation of endothelin production |
|  | GO:0051602 | positive regulation of endothelin production |
|  | GO:0050953 | regulation of multicellular organismal development |
|  | GO:0050905 | response to stimulus |
|  | GO:0050890 | regulation of multicellular organismal process |
|  | GO:0050877 | neuron apoptotic process |
|  | GO:0048869 | neuron death |
|  | GO:0048856 | sodium ion homeostasis |
|  | GO:0048731 | cellular component organization or biogenesis |
|  | GO:0042745 | synaptic signaling |
|  | GO:0042063 | synaptic transmission, dopaminergic |
|  | GO:0035809 | cell-cell signaling |
|  | GO:0031223 | synaptic transmission, cholinergic |
|  | GO:0031175 | p38MAPK cascade |
|  | GO:0030431 | synaptic transmission, GABAergic |
|  | GO:0030154 | ERK1 and ERK2 cascade |
|  | GO:0022008 | regulation of long-term synaptic potentiation |
|  | GO:0021769 | neurotransmitter uptake |
|  | GO:0014009 | desensitization of G protein-coupled receptor signaling pathway |
|  | GO:0014002 | regulation of G protein-coupled receptor signaling pathway |
|  | GO:0010996 | adaptation of signaling pathway |
|  | GO:0009612 | regulation of defense response |
|  | GO:0007638 | regulation of synaptic transmission, cholinergic |
|  | GO:0007631 | positive regulation of synaptic transmission, cholinergic |
|  | GO:0007622 | regulation of synaptic transmission, GABAergic |
|  | GO:0007611 | regulation of renal sodium excretion |
|  | GO:0007601 | regulation of excretion |
|  | GO:0007588 | negative regulation of synaptic transmission |
|  | GO:0007416 | positive regulation of synaptic transmission |
|  | GO:0007399 | regulation of neurotransmitter uptake |
|  | GO:0007275 | positive regulation of dopamine uptake involved in synaptic transmission |
|  | GO:0003008 | regulation of neurotransmitter transport |
|  | GO:0002093 | positive regulation of amine transport |
|  | GO:0002041 | long-term synaptic depression |
|  | GO:0001964 | regulation of ERK1 and ERK2 cascade |
|  | GO:0007610 | regulation of response to stress |
| regulation of long-term synaptic potentiation | GO:1900744 | protein dephosphorylation |
|  | GO:1900453 | dephosphorylation |
|  | GO:1900452 | protein metabolic process |
|  | GO:0099177 | organonitrogen compound metabolic process |
|  | GO:0098801 | endothelin production |
|  | GO:0080134 | behavior |
|  | GO:0070372 | startle response |
|  | GO:0060292 | intussusceptive angiogenesis |
|  | GO:0051954 | auditory receptor cell morphogenesis |
|  | GO:0051588 | system process |
|  | GO:0051586 | multicellular organism development |
|  | GO:0051580 | nervous system development |
|  | GO:0050806 | synapse assembly |
|  | GO:0050805 | excretion |
|  | GO:0044062 | visual perception |
|  | GO:0035813 | learning or memory |
|  | GO:0032228 | rhythmic behavior |
|  | GO:0032224 | feeding behavior |
|  | GO:0032222 | mechanosensory behavior |
|  | GO:0031347 | response to mechanical stimulus |
|  | GO:0023058 | response to auditory stimulus |
|  | GO:0008277 | astrocyte development |
|  | GO:0002029 | glial cell proliferation |
|  | GO:0001504 | orbitofrontal cortex development |
|  | GO:1900271 | neurogenesis |
| response to purine-containing compound | GO:1901698 | positive regulation of programmed cell death |
|  | GO:0051591 | regulation of kinase activity |
|  | GO:0046683 | negative regulation of phosphorus metabolic process |
|  | GO:0043279 | regulation of protein modification process |
|  | GO:0043278 | positive regulation of protein modification process |
|  | GO:0035094 | regulation of phosphorus metabolic process |
|  | GO:0014075 | regulation of transferase activity |
|  | GO:0014070 | regulation of CDP-diacylglycerol-serine O-phosphatidyltransferase activity |
|  | GO:0010243 | positive regulation of CDP-diacylglycerol-serine O-phosphatidyltransferase activity |
|  | GO:0010033 | positive regulation of serine C-palmitoyltransferase activity |
|  | GO:0009719 | regulation of protein geranylgeranylation |
|  | GO:0009628 | positive regulation of protein geranylgeranylation |
|  | GO:0001975 | catecholamine secretion |
|  | GO:0014074 | regulation of behavior |
| regulation of behavior | GO:2000026 | sensory perception of light stimulus |
|  | GO:1904472 | response to electrical stimulus |
|  | GO:1904470 | general adaptation syndrome |
|  | GO:1900168 | general adaptation syndrome, behavioral process |
|  | GO:1900166 | linear vestibuloocular reflex |
|  | GO:0051962 | dendritic cell differentiation |
|  | GO:0051240 | cell death |
|  | GO:0045887 | cell population proliferation |
|  | GO:0045187 | negative regulation of cell population proliferation |
|  | GO:0032731 | negative regulation of locomotion |
|  | GO:0010625 | positive regulation of monocyte extravasation |
|  | GO:0050795 | response to purine-containing compound |
| regulation of kinase activity | GO:2000541 | response to abiotic stimulus |
|  | GO:2000539 | response to endogenous stimulus |
|  | GO:1904222 | response to organic substance |
|  | GO:1904219 | response to organonitrogen compound |
|  | GO:1904217 | response to organic cyclic compound |
|  | GO:0051338 | response to amine |
|  | GO:0051174 | response to nicotine |
|  | GO:0031401 | response to morphine |
|  | GO:0031399 | response to alkaloid |
|  | GO:0010563 | response to organophosphorus |
|  | GO:0043549 | response to cAMP |
| synaptic signaling | GO:0070371 | cell differentiation |
|  | GO:0051932 | sleep |
|  | GO:0038066 | neuron projection development |
|  | GO:0007271 | auditory behavior |
|  | GO:0007267 | regulation of urine volume |
|  | GO:0001963 | gliogenesis |
|  | GO:0099536 | circadian sleep/wake cycle |
| protein dephosphorylation | GO:1990775 | regulation of renal system process |
|  | GO:1901564 | regulation of trans-synaptic signaling |
|  | GO:0019538 | regulation of long-term synaptic depression |
|  | GO:0016311 | negative regulation of long-term synaptic depression |
|  | GO:0006470 | regulation of p38MAPK cascade |
| regulation of neuron apoptotic process | GO:0043068 | response to nitrogen compound |
|  | GO:0043065 | reproductive process |
|  | GO:0043523 | cell projection organization |
| negative regulation of cell population proliferation | GO:2000439 | positive regulation of Schwann cell proliferation |
|  | GO:0040013 | positive regulation of interleukin-1 beta production |
|  | GO:0008285 | regulation of circadian sleep/wake cycle, sleep |
| neuron apoptotic process | GO:0070997 | cellular developmental process |
|  | GO:0051402 | nervous system process |
| cell projection organization | GO:0016043 | regulation of programmed cell death |
|  | GO:0030030 | regulation of neuron apoptotic process |
| - | GO:1901214 | reproduction |
| - | GO:0071840 | system development |
| - | GO:0055078 | anatomical structure development |
| - | GO:0051239 | cognition |
| - | GO:0050896 | neuromuscular process |
| - | GO:0050432 | response to amphetamine |
| - | GO:0043067 | cellular component organization |
| - | GO:0042127 | multicellular organismal process |
| - | GO:0032502 | developmental process |
| - | GO:0032501 | regulation of cell population proliferation |
| - | GO:0022414 | positive regulation of apoptotic process |
| - | GO:0008283 | positive regulation of synaptic assembly at neuromuscular junction |
| - | GO:0008219 | positive regulation of multicellular organismal process |
| - | GO:0000003 | regulation of neuron death |

Supplementary Table 3. Specified GOs in each category of the hippocampus.

| Category | Gene Ontology ID | Gene Ontology Description |
| --- | --- | --- |
| response to cytokine | GO:0140546 | defense response to symbiont |
|  | GO:0071345 | cellular response to cytokine stimulus |
|  | GO:0071310 | cellular response to organic substance |
|  | GO:0070887 | cellular response to chemical stimulus |
|  | GO:0070301 | cellular response to hydrogen peroxide |
|  | GO:0060337 | type I interferon-mediated signaling pathway |
|  | GO:0051607 | defense response to virus |
|  | GO:0042538 | hyperosmotic salinity response |
|  | GO:0042221 | response to chemical |
|  | GO:0035634 | response to stilbenoid |
|  | GO:0035458 | cellular response to interferon-beta |
|  | GO:0035457 | cellular response to interferon-alpha |
|  | GO:0035456 | response to interferon-beta |
|  | GO:0035455 | response to interferon-alpha |
|  | GO:0034340 | response to type I interferon |
|  | GO:0010035 | response to inorganic substance |
|  | GO:0010033 | response to organic substance |
|  | GO:0009746 | response to hexose |
|  | GO:0009615 | response to virus |
|  | GO:0009607 | response to biotic stimulus |
|  | GO:0009605 | response to external stimulus |
|  | GO:0006952 | defense response |
|  | GO:0006950 | response to stress |
|  | GO:0034097 | response to cytokine |
| tube morphogenesis | GO:0072359 | circulatory system development |
|  | GO:0050891 | multicellular organismal-level water homeostasis |
|  | GO:0048869 | cellular developmental process |
|  | GO:0048856 | anatomical structure development |
|  | GO:0048731 | system development |
|  | GO:0048646 | anatomical structure formation involved in morphogenesis |
|  | GO:0048513 | animal organ development |
|  | GO:0035295 | tube development |
|  | GO:0030154 | cell differentiation |
|  | GO:0019228 | neuronal action potential |
|  | GO:0009888 | tissue development |
|  | GO:0009653 | anatomical structure morphogenesis |
|  | GO:0007275 | multicellular organism development |
|  | GO:0007166 | cell surface receptor signaling pathway |
|  | GO:0001944 | vasculature development |
|  | GO:0001885 | endothelial cell development |
|  | GO:0001816 | cytokine production |
|  | GO:0035239 | tube morphogenesis |
| negative regulation of viral process | GO:2000026 | regulation of multicellular organismal development |
|  | GO:1903999 | negative regulation of eating behavior |
|  | GO:0070169 | positive regulation of biomineral tissue development |
|  | GO:0051240 | positive regulation of multicellular organismal process |
|  | GO:0051094 | positive regulation of developmental process |
|  | GO:0048523 | negative regulation of cellular process |
|  | GO:0010628 | positive regulation of gene expression |
|  | GO:0001819 | positive regulation of cytokine production |
|  | GO:0001817 | regulation of cytokine production |
|  | GO:0048525 | negative regulation of viral process |
| secretion | GO:1990961 | xenobiotic detoxification by transmembrane export across the plasma membrane |
|  | GO:0055085 | transmembrane transport |
|  | GO:0042908 | xenobiotic transport |
|  | GO:0042886 | amide transport |
|  | GO:0034220 | monoatomic ion transmembrane transport |
|  | GO:0006810 | transport |
|  | GO:0002790 | peptide secretion |
|  | GO:0046903 | secretion |
| regulation of cytokine-mediated signaling pathway | GO:0060759 | regulation of response to cytokine stimulus |
|  | GO:0045088 | regulation of innate immune response |
|  | GO:0043410 | positive regulation of MAPK cascade |
|  | GO:0043408 | regulation of MAPK cascade |
|  | GO:0031347 | regulation of defense response |
|  | GO:0002831 | regulation of response to biotic stimulus |
|  | GO:0001959 | regulation of cytokine-mediated signaling pathway |
| extracellular matrix organization | GO:0097435 | supramolecular fiber organization |
|  | GO:0051764 | actin crosslink formation |
|  | GO:0045229 | external encapsulating structure organization |
|  | GO:0043062 | extracellular structure organization |
|  | GO:0030036 | actin cytoskeleton organization |
|  | GO:0030198 | extracellular matrix organization |
| regulation of hydrolase activity | GO:0097202 | activation of cysteine-type endopeptidase activity |
|  | GO:0050790 | regulation of catalytic activity |
|  | GO:0051336 | regulation of hydrolase activity |
| positive regulation of fibroblast proliferation | GO:0040017 | positive regulation of locomotion |
|  | GO:0008284 | positive regulation of cell population proliferation |
|  | GO:0048146 | positive regulation of fibroblast proliferation |
| icosanoid biosynthetic process | GO:0034638 | phosphatidylcholine catabolic process |
|  | GO:0046456 | icosanoid biosynthetic process |
| - | GO:0065009 | regulation of molecular function |
| - | GO:0051239 | regulation of multicellular organismal process |
| - | GO:0051179 | localization |
| - | GO:0051046 | regulation of secretion |
| - | GO:0050896 | response to stimulus |
| - | GO:0050793 | regulation of developmental process |
| - | GO:0050792 | regulation of viral process |
| - | GO:0050673 | epithelial cell proliferation |
| - | GO:0044419 | biological process involved in interspecies interaction between organisms |
| - | GO:0042127 | regulation of cell population proliferation |
| - | GO:0032502 | developmental process |
| - | GO:0032501 | multicellular organismal process |
| - | GO:0030029 | actin filament-based process |
| - | GO:0019886 | antigen processing and presentation of exogenous peptide antigen via MHC class II |
| - | GO:0019058 | viral life cycle |
| - | GO:0016032 | viral process |
| - | GO:0008283 | cell population proliferation |
| - | GO:0007155 | cell adhesion |
| - | GO:0006915 | apoptotic process |
| - | GO:0002376 | immune system process |
